# Heat penetration attributes of milkfish (*Chanos chanos*) thermal processed in flexible pouches: a comparative study between steam application and water immersion

**DOI:** 10.1002/fsn3.426

**Published:** 2016-10-02

**Authors:** Mary A. Adepoju, Bamidele O. Omitoyin, Chitradurga O. Mohan, Aliyam A. Zynudheen

**Affiliations:** ^1^ Food Technology DepartmentFederal Institute of Industrial ResearchOshodiLagosNigeria; ^2^ Department of Fisheries and AquacultureUniversity of IbadanIbadanNigeria; ^3^ Fish Processing DivisionCentral Institute of Fisheries TechnologyCochinIndia

**Keywords:** *F*_0_, flexible pouches, heat penetration, milkfish, steam–air, water immersion

## Abstract

The difference in the heating penetration characteristics of product processed in retort by steam–air application and water immersion was studied. Fresh milkfish (*Chanos chanos*) packed in dry pack and in oil medium, both in flexible pouches, was thermal processed to minimum *F*_0_ value of 7.77 at 121.1°C. Heat penetration values were recorded for each minute of processing with the aid Ellab (TM 9608, Denmark) temperature recorder. Retort come up time to achieve 121.1°C was observed to be less in steam–air which invariably led to a lower Ball's process time (B) and the total process time (T) observed in steam–air as compared to water immersion. Obtained data were plotted on a semi‐logarithmic paper with temperature deficit on *x*‐axis against time on the *y*‐axis.

## 1 Introduction

One of the commonest and the most important technique employed in the food industry to extend the shelf‐life of foods is thermal processing. It majorly requires heating of foods for a scheduled time at a preselected temperature with the aim of eliminating pathogenic microorganisms of public health importance as well as those microorganisms and enzymes that deteriorate food during storage. These days, the consumers demand is more for high‐quality foods than the production of safe and shelf‐stable foods. The severity of the thermal process determines the degradation of food quality in terms of the sensory (color, flavor, texture) and nutritional factors (Smout, Avila, Van Loey, Hendrickx, & Silva, 2000).

Use of retort pouch in fish processing serves the dual purpose of packaging and processing as freshly packed fish is cooked during the processing line. It employs sterilization technique during processing. In commercial practice, the sterilization of food is determined to have been accomplished after the food has been packed inside a hermetically sealed container. In the findings of Smout et al. (2000), it was reported that there are two main factors contributing to the nature of nonuniformity of safety and quality during thermal processing of food which are (1) the variability in heat delivery by the thermal process equipment to the food surface (heat distribution) and (2) variability in heat delivery from the surface to the coldest spot of the food product (heat penetration).

Products packaged and processed in flexible pouches need significantly less heat compared to cans in order to achieve commercial sterility; cooking time and energy costs are therefore reduced by half (Jun et al., 2006). It is gradually becoming a preferred technology over canning in the international market that is satisfying the crave for ready to eat food (RTE‐F). As the century grows, most people tend to live their lives on the go and this has resulted in the crave for ready to eat food (RTE‐F).

Different versions of retort systems with their operations have been described previously (Lampi, 1977; Venugopal, 2006; Yamaguchi, 1990). Steam–air retort system and water immersion over pressure retort systems are usually used for thermal processing of food in flexible pouches. Water immersion retort is the type where processing water is first heated in a separate vessel before being pumped into the processing vessel once the water reaches the desired processing temperature. Usually, sufficient water to completely cover the packages is used and the water is recirculated all through the processing period. But, in some other cases, packages are only partially covered with water during processing. In steam–air application, steam and air are continuously passed through the vessel to create a homogeneous mixture throughout the retort. This generates an overpressure condition in the retort and lead to continuous venting of the steam/air mixture thereby creating constant flow of steam past the containers. Degree of heat penetration in canned foods is affected by so many factors such as retort temperature, heat transfer medium, process time, product consistency, and the method of packing the products among others. At the National Canners’ Association in Washington, some work was carried out by Bigelow, Bohart, Richardson, and Ball (1920) on heat penetration of canned foods and the findings were published. So many works have been done on the use of retort technology, but not much has been done in comparing the rate of heat penetration in the different methods employed in retort technology. Hence, this work was carried out to compare the heat penetration rate of fresh milkfish subjected to steam application and water immersion which seems to be the most common methods used in retort technology.

## 2 Materials and Methods

### 2.1 Materials

Fresh milkfish (*Chanos chanos*) used for the study was obtained from a farm in Kochi, Kerala, India, where they were cultured under brackish water condition and transported to the laboratory in chilled condition. Other materials used are refined sunflower oil, salt, and four‐layered opaque aluminum foil flexible pouches. All the materials were procured from Cochin local market.

### 2.2 Sample preparation

The fish were immediately dressed (cleaned, gutted, scale removed, and washed), cut into steaks of 3 cm size, and brined in 2% chilled solution in the ratio of 1:1 (w/v) for 10 min.

The steaks were filled into the pouches in 200 g of fish per pouch for the dry pack and 120 g fish to 80 ml of oil for the oil medium. Before filling two of the pouches, a thermocouple was fixed at the base of each one to record the core temperature. The pouches for the dry pack were precooked in steam for 5 min at 100°C after which they were drained of water, then immediately vacuum sealed for proper air removal. The oil medium pouches were filled with 80 ml of hot sunflower oil and sealed immediately. The pouches were loaded into the retort and thermal processed to *F*_0_ value of 8 at 121.1°C. The minimum *F*_0_ value (lethality) required for canned food product is usually 2.52 min at 121.1°C, to inactivate spores of *Clostridium botulinum*. The recommended *F*_0_ values for fish and fishery products range between 5 and 20 min (Bratt, 1995; Frott & Lewis, 1994; Pflug & Christensen, 1980). Therefore, an *F*_0_ value of 8.0 min was selected for this study as recommended previously (Frott & Lewis, 1994; Gopal, Vijayan, Balachandran, Madhavan, & Iyer, 2001; Mohan, Remya, Murthy, Ravishankar, & Asok, 2015).

Heat penetration values were recorded for every single minute of processing using Ellab (TM 9608, Denmark) temperature recorder. After thermal processing, the pouches were immediately cooled to a core temperature of maximum of 60°C by releasing potable water into the retort. Obtained data were plotted on a semi‐logarithmic paper with temperature deficit on *x*‐axis and time on the *y*‐axis. The lag factor for heating (Jh), slope of the heating curve (fh), *F*_0_ value (lethality), time recorded in minutes for sterilization at retort temperature (U), lag factor for cooling (jc), final temperature deficit (g), and cook value (cg) were determined. Ball's process time (B) was calculated and the actual process time, which is the total process time, was determined by adding total process time (T) and 58% of the come up time (CUT).

### 2.3 Statistical analysis

The obtained data were statistically analyzed using version 10.00 of Statistical Product and Service Solution (SPSS) (SPSS, 2000). Analysis of variance was used to calculate significant difference between the two processing methods employed. Differences were considered to be significant at *p *<* *.05 using *t* test at a 95% confidence level.

## 3 Results and Discussion

**Figure 1:**
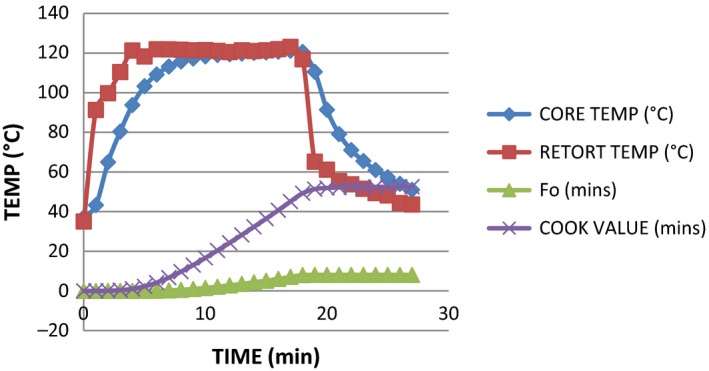
Graph showing the heat penetration attributes (*F*_0_ value, cook value, retort temperature, and core temperature) of milkfish thermal processed by steam–air in dry pack based on temperature (°C) and time (minutes). Core temperature at the beginning and at the end of processing = 37.08°C and 51°C, respectively. Retort temperature at the beginning and at the end of processing = 35.02°C and 43.58°C, respectively. Lethality (*F*_0_) 8.04 and cook value 52.55 were achieved at 27 min

**Figure 2:**
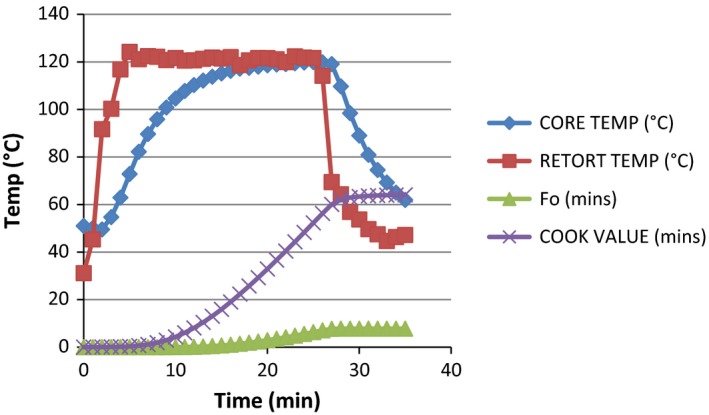
Graph showing the heat penetration attributes (*F*_0_ value, cook value, retort temperature, and product temperature) of milkfish thermal processed by steam–air in oil medium based on temperature (°C) and time (minutes). Core temperature at the beginning and at the end of processing = 51°C and 61.94°C, respectively. Retort temperature at the beginning and at the end of processing = 31.14°C and 47.2°C, respectively. Lethality (*F*_0_) 7.87 and cook value 64.09 were achieved at 35 min

**Figure 3:**
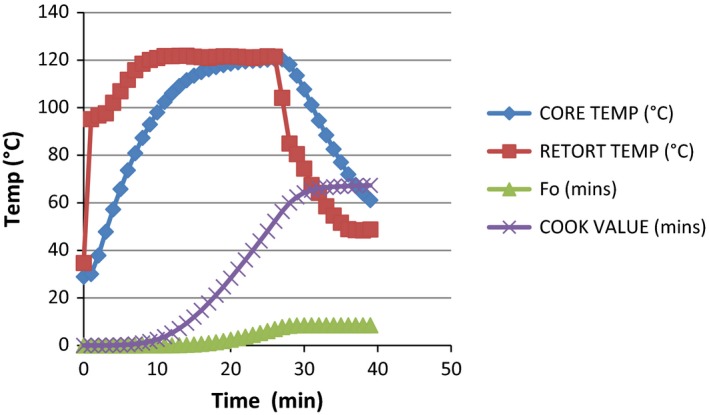
Graph showing the heat penetration attributes (*F*_0_ value, cook value, retort temperature, and product temperature) of milkfish thermal processed by water immersion in dry pack based on temperature (°C) and time (minutes). Core temperature at the beginning and at the end of processing = 28.89°C and 61.14°C, respectively. Retort temperature at the beginning and at the end of processing = 34.67°C and 48.77°C, respectively. Lethality (*F*_0_) 8.49 and cook value 67.32 were achieved at 39 min

**Figure 4:**
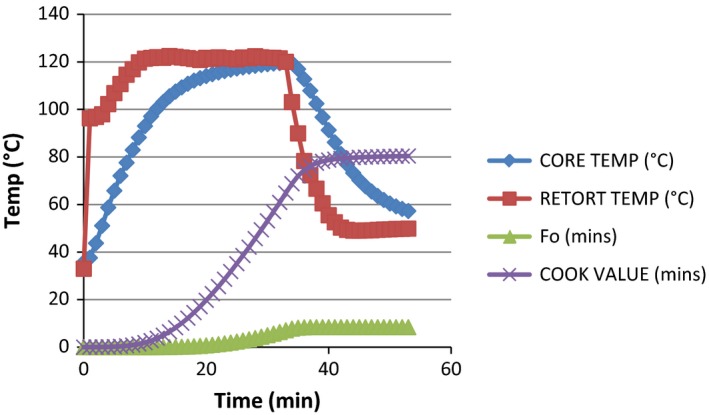
Graph showing the heat penetration attributes (*F*_0_ value, cook value, retort temperature, and product temperature) of milkfish thermal processed by water immersion in oil medium based on temperature (°C) and time (minutes). Core temperature at the beginning and at the end of processing = 35.7°C and 57.27°C, respectively. Retort temperature at the beginning and at the end of processing = 33°C and 49.88°C, respectively. Lethality (*F*_0_) 8.41 and cook value 80.43 were achieved at 53 min

Result of the effect of the two sterilization methods (steam–air and water immersion) on the heat penetration attributes of fresh milkfish processed in oil medium and dry pack is presented in Table 1. The graphs showing the *F*_0_ value, cook value, retort temperature, and product temperature are given in Figs 1, 2, 3, 4.

**Table 1:** Heat penetration attributes of fresh milkfish thermal processed by steam–air application and water immersion

Parameters	Steam–air		Water immersion	
	Dry pack	Oil medium	Dry pack	Oil medium
CUT	4^a^	6^b^	11^d^	10^c^
fh (min)	6^a^	11^b^	11^b^	17^c^
Jh	0.52^c^	0.88^d^	0.50^a^	0.56^b^
jc	0.68^a^	0.99^d^	0.93^c^	0.93^b^
G	0.12^c^	0.58^a^	0.49^b^	1.09^d^
*F*_0_	8.04^b^	7.77^a^	8.49^d^	8.41^c^
B (min)	15.34^a^	22.34^c^	21.72^d^	27.97^b^
T_B_ (min)	17.66^a^	25.82^b^	28.1^c^	33.77^d^
cg (min)	52.56^a^	64.09^b^	67.32^c^	80.43^d^

CUT, come up time.

^abcd^Values arranged in the same row and followed by different letters are significantly different at *p* < .05.

The CUT describes the time taken for the retort to attain the required temperature of 121.1°C was observed to be faster in steam application at 4 and 6 min than water immersion at 11 and 10 for the dry pack and oil medium pack, respectively. In retort processing, the time taken for a heat penetration curve to traverse one log cycle is called the heating rate index fh value. Heating rate index (fh) for convective heating is a factor dependent on temperature of the heating medium as well as the condensing surface in terms of surface size and orientation. This explains the high fh value in the oil pack medium of both the steam application and water immersion (Table 1), considering the use of hot oil as a medium.

In foods processed in cans, the lag factor for heating (Jh) is related to the lag time required to reach harmonized heating rate values according to Mohan et al. (2015). Mohan et al. (2015) reported that at the completion of the heating process, water is pumped into the retort for cooling process to start, there is a lag before cooling water begins to lower the product temperature (Jc) resulting in a significant amount of heating even after the steam has been turned off. Heating lag factor (Jh) that is close to or above1.0 and cooling lag factor (Jc) less than 1.0 is an indication of faster heat penetration and this is evident in steam–air with Jh 0.88 compared to 0.56 of water immersion. Heating lag factor (Jh) in both methods were less than 1.0 with minimal lag period (i.e., the come up time is very short); therefore, PID and ID overlap, so that Jh is equal to 1.0 (Jones, 1968). Higher values of Jh of 1.44 were observed in retort pouch‐processed prawn kruma and 1.0 for can‐processed prawn kruma, respectively (Mohan et al., 2008). Cook value (cg) refers to the value that gives a suggestion of the impact of thermal processing on food with respect to nutrient degradation and it should be as minimal as possible at any given lethality (Mohan et al., 2015). In this study, the minimum cg was observed in steam–air application with 52.56 and 64.09 min in the dry and oil medium pack, respectively, as against 67.32 and 80.43 min for dry and oil medium pack in water immersion application.

The total process time (T_B_) for fresh milkfish in dry pack was observed to be 17.66 min to achieve *F*_0_ value of 8.04 in steam application and 28.1 for *F*_0_ value of 8.49 in water immersion, while the T_B_ for the milkfish packed in oil medium was 25.82 at *F*_0_ value of 7.77 in steam and 33.77 at 8.41 in water immersion. The least process time observed in steam application can be attributed to the fast come up time in the method which resulted into faster heating rate. This actually conforms with the finding of Mohan et al. (2015), which explained that the least process time observed for tuna with broccoli pack could be attributed to soft nature of broccoli, which resulted in the faster heating rate. These findings indicate that the relationship between the retort CUT and the T_B_ is directly proportional. From the data generated and from the graph in Figs 1, 2, 3, 4, it was observed that the rate of heat penetration was faster in steam application than in water immersion. The Tukey's test performed on the heat penetration values obtained for the two processing methods showed significant differences (*p *<* *.05). However, the heating rate indices of the samples processed in oil medium by steam application and in the dry pack by water immersion are not significantly different (*p *>* *.05).

The difference in the heat penetration rate can be attributed to the mode of heat transfer in the two methods. While heat transfer in water immersion is by conduction, it is a combination of both convection and conduction in steam–air application. Heat transfer in convection occurs by the movement of air, liquid, or steam around the food, whereas it is by direct physical contact in conduction. The efficiency of the heat transfer in conduction depends on the conductivity of materials in contact with the food, while the efficiency in convection is by circulation of air, thereby making heat transfer faster in the latter.

## 4 Conclusion

Minimum CUT led to faster heating rate which decreased total process time in steam–air retort. Cook value was also low in steam–air. Although the heat penetration data generated for the two methods of sterilization are within acceptable range required to achieve good product quality, steam–air retort has an advantage over water immersion because of its faster rate of heat penetration and reduced process time; the lower the process time, the better the nutritional and sensory quality of a product. Further studies to compare the degree of heat penetration in both steam retort system and water immersion retort system are necessary in order to increase the knowledge base on the degree of heat penetration in the two methods.

## Funding Information

This work was supported by the Centre for Science and Technology of the Non‐Aligned and other Developing Countries (NAM S&T) for research fellowship at the Central Institute of Fisheries Technology, Kochi, Kerala, India.

## Conflict of Interest

None declared.
